# Annual Report of the Smithsonian Institution, for 1899

**Published:** 1902-03

**Authors:** 


**Annual Report of the Smithsonian Institution, for 1899.** Washington: Government Printing Office. 1901.

The collection of essays forming the general appendix of this annual report is always of most absorbing interest; and the volume before us, containing for example papers on Liquid Hydrogen, by Prof. Dewar; Psychical Research, by Sir W. Crookes; the Petrified Forests of Arizona, by Lester F. Ward, &c., &c., is if possible more fascinating than usual, and when once begun the volume must be read from cover to cover.

Even the secretary's business report, which occupies the first 87 pages, is worth reading. How long will it be, we wonder, before the Secretary of our Royal Society is empowered by the Admiralty and War Office to issue circulars to officers on foreign service to make "such collections of rare animals as were practicable for the national zoological collection," this circular containing at the same time directions for shipping and feeding any animals that may be collected? Also, to diplomatic and consular officers to report on any important antiquarian discoveries that may be made in their districts. They do these things better in America.

